# Comparison of spontaneous brain activity in distinguishing parkinsonian variant of multiple system atrophy from Parkinson’s disease at an early stage

**DOI:** 10.3389/fnagi.2024.1427991

**Published:** 2024-08-29

**Authors:** Shichan Wang, Yi Xiao, Yanbing Hou, Chunyu Li, Lingyu Zhang, Ruwei Ou, Qianqian Wei, Junyu Lin, Tianmi Yang, Ningning Che, Qirui Jiang, Xiaoting Zheng, Jiyong Liu, Huifang Shang

**Affiliations:** ^1^Laboratory of Neurodegenerative Disorders, National Clinical Research Center for Geriatrics, West China Hospital, Department of Neurology, Sichuan University, Chengdu, China; ^2^National Clinical Research Center for Geriatrics (WCH), West China Hospital, Sichuan University, Chengdu, China; ^3^Center of Gerontology and Geriatrics, West China Hospital, Sichuan University, Chengdu, China

**Keywords:** multiple system atrophy, Parkinson’s disease, early-stage, resting-state functional magnetic resonance imaging, fractional amplitude of low-frequency fluctuation

## Abstract

**Background:**

The overlapping clinical manifestations in parkinsonian variant of multiple system atrophy (MSA-P) and Parkinson’s Disease (PD) can complicate clinical diagnostic accuracy, particularly in the early stage. The study aims to uncover the patterns of brain function in the initial phase of the two conditions.

**Methods:**

We recruited 24 MSA-P patients, 34 PD patients and 27 healthy controls (HC). Voxel-wise fractional amplitude of low-frequency fluctuation (fALFF) was compared to characterize regional brain function, followed by seed-based functional connectivity (FC) analysis. Receiver operating characteristic (ROC) analyses were used to examine the diagnostic accuracy of fALFF.

**Results:**

Compared to HC, decreased fALFF was observed in the bilateral basal ganglia (BG) of MSA-P patients, while decreased fALFF was identified in the left BG of PD patients. Additionally, elevated fALFF was found in the superior cerebellum for MSA-P patients and the temporo-occipital cortex for PD patients. Furthermore, PD patients exhibited increased FC in the cortico-striatal loop compared to MSA-P patients. The fALFF of the left caudate distinguished MSA-P from HC with an area under the curve (AUC) of 0.838 (*p* < 0.001) and from PD with an AUC of 0.772 (*p* < 0.001). The fALFF of the left putamen distinguished PD from HC with an AUC of 0.736 (*p* = 0.002).

**Conclusion:**

Our findings indicated common and distinct abnormalities in spontaneous brain activity within BG, cerebellum, and cortices in early-stage MSA-P and PD patients. PD patients employed more compensatory mechanisms than MSA-P patients. Furthermore, fALFF may aid in early differentiation between MSA-P and PD.

## Introduction

Parkinson disease (PD) is the second-most common neurodegenerative disorder, primarily characterized by movement symptoms, accompanied by a range of non-motor features, such as sleep disorders, depression, cognitive impairment, and autonomic dysfunction (Poewe et al., 2017). With the global population aging, there is an anticipated increase in the societal and economic burden associated with the disease (Ben-Shlomo et al., 2024). The pathological features of PD include the degeneration of dopaminergic neurons in the nigrostriatal pathway and the formation of intracellular inclusion bodies containing aggregated misfolded α-synuclein protein (Haider et al., 2023), known as α-synucleinopathy. Multiple system atrophy (MSA), as another α-synucleinopathy, is a rare neurodegenerative disease manifesting with a combination of autonomic failure, parkinsonism or ataxia, and characterized by oligodendroglial cytoplasmic inclusions containing abnormally aggregated α-synuclein (Poewe et al., 2022). Patients with MSA displaying predominant parkinsonism symptoms and cerebellar syndrome are classified as the MSA-P subtype and MSA-C subtype, respectively (Wenning et al., 2022). However, compared to patients with PD, MSA-P patients often exhibit poor levodopa responsive parkinsonism, a more aggressive course, and severer disability. Due to phenotypic similarities and the lack of definitive tests or biomarkers in the early stage, the clinical diagnostic accuracy of PD and MSA-P remains challenging, leading to delays or even the absence of precise patient identification and intervention (Tolosa et al., 2021; Wenning et al., 2022). Therefore, it is imperative to identify effective biomarkers for differentiation at an early stage.

Various imaging modalities can evaluate patterns of brain function activities (Wise, 2013), and were widely employed in identifying neurodegenerative diseases, depicting disease trajectories, and providing insights into underlying mechanisms (Young et al., 2024). Among them, resting-state functional magnetic resonance imaging (rs-fMRI), achieved by tracking fluctuations in local blood oxygen-level dependent signals, stands out as a non-invasive and accessible technique, gradually demonstrating significant value in the diagnosis and treatment of neurodegenerative disorders (Perovnik et al., 2023). Most fMRI studies concentrated on correlations between different brain regions or networks in terms of functional connectivity (FC), without directly revealing the amplitude of brain activity in each region. For instance, several studies investigating both motor and non-motor symptoms in PD patients employed the seed-based approach, according to the pathological mechanisms of PD, focusing on the disturbance in FC associated with the striatum (putamen and caudate nucleus) (Cerasa et al., 2016). Patients with PD often displayed disrupted FC between these seeds and various subcortical and cortical regions (Wu et al., 2015). However, the assessment of changes in FC between specific areas yielded inconsistent results due to variations in disease duration, medication status, subtypes and sample sizes of PD patients across studies. As a supplement, the amplitude of low-frequency fluctuation (ALFF) of the rs-fMRI signal was proposed to assess the intensity of regional spontaneous brain activity. The fractional ALFF (fALFF) method was further introduced to enhance sensitivity and specificity in identifying these activities compared to ALFF (Zou et al., 2008). In a recent study, Hou et al. discovered that models based on fALFF values might contribute to distinguishing PD patients from healthy controls (HC) and predicting future deterioration (Hou et al., 2022).

Considering the clinical significance of early identification for MSA-P and PD patients, along with the heterogeneity in previous research and the potential discriminatory value of the fALFF, therefore, the current study was conducted. The primary objective was to explore distinctions in fALFF indicator among early-stage MSA-P and PD patients. Additionally, we sought to investigate variations in FC between the two diseases and evaluate the diagnostic and discriminatory utility of fALFF for the two conditions.

## Materials and methods

### Subjects

Participants in the study were recruited from an ongoing prospective longitudinal cohort study at the Department of Neurology, Sichuan University West China Hospital. The study received approval from the Ethics Committee of West China Hospital, Sichuan University (No. 2015–236), and all participants provided written informed consent. Diagnosis of PD and MSA was based on the Movement Disorder Society clinical diagnostic criteria for PD (MDS-PD Criteria) (Postuma et al., 2015) and the Movement Disorder Society Criteria for the Diagnosis of Multiple System Atrophy (MDS-MSA Criteria) (Wenning et al., 2022), respectively. Patients were categorized into MSA-P subtype based on the predominant symptoms of parkinsonism at the time of evaluation. Each patient underwent evaluation by at least two neurologists specializing in movement disorders and was followed up at least once through telephone or face-to-face interviews. The last visits were scheduled for December 2023. Exclusion criteria included (1) left-handedness; (2) Hoehn & Yahr (H&Y) stage >2.5 or Unified Multiple System Atrophy Rating Scale-I (UMSARS-I) ≥ 17; (3) disease duration >3 years; (4) a history of other neurological or psychiatric diseases, head injury, and neurological surgery; (5) visible structural brain defects. Age- and sex-matched HC were included if they were (1) right-handed; (2) had no history of neurological or psychiatric diseases; (3) had no family history of PD; (4) had normal brain structure.

Demographic and clinical information, including age, sex, education level, disease duration and medication usage, was collected from patients. Motor symptom severity in PD patients was assessed through the MDS-Unified PD Rating Scale (MDS-UPDRS) Part III and H&Y stage (Goetz et al., 2008; Hoehn and Yahr, 1967), whereas, it was assessed by using Part II of the UMSARS in MSA-P patients (Wenning et al., 2004), during the OFF-state after instructing patients to withhold anti-parkinsonian drugs for at least 12 h. Depression and anxiety were evaluated by using the 24-item Hamilton Depression Rating Scale (HAMD) (Moberg et al., 2001) and the Hamilton Anxiety Rating Scale (HAMA) (Hamilton, 1959), respectively. Cognitive function was assessed by using the Montreal Cognitive Assessment (MoCA) (Nasreddine et al., 2005).

### MRI acquisition and preprocessing

All MRI scans were conducted using a 3.0 Tesla MRI scanner (Magnetom Skyra; Siemens Healthineers, Erlangen, Germany) equipped with a 32-channel head coil. The rs-fMRI data were obtained through a gradient-echo echo-planar imaging sequence with the following parameters: repetition time (TR) = 2000 ms, echo time (TE) = 30 ms, flip angle = 90°, field of view (FOV) = 224 × 224 mm^2^, matrix size = 64 × 64, slice number = 36, slice thickness = 3.5 mm, slice gap = 0.7 mm, and voxel size = 3.5 × 3.5 × 3.5 mm^3^. Participants were instructed to lie comfortably in supine position with closed eyes. The foam pad minimized head movement, and earplugs were used to reduce noise interference. Participants were routinely checked to ensure wakefulness.

The rs-fMRI data underwent preprocessing using the Statistical Parametric Mapping software (SPM12, https://www.fil.ion.ucl.ac.uk) and Data Processing & Analysis for Brain Imaging toolkit (DPABI, http://rfmri.org/DPABI) (Chao-Gan and Yu-Feng, 2010). Preprocessing steps included removing the first 10 time points, slice timing correction, motion correction, spatial normalization to the standard Montreal Neurological Institute (MNI) EPI templates, resampling to 3 × 3 × 3 mm^3^, spatial smoothening with 4-mm full-width at half-maximum (FWHM) Gaussian kernel, detrending, and nuisance signal regression (Friston 24-parameters, white matter, and cerebrospinal fluid signals). Head motion parameters for all participants were limited to <2 mm maximum displacement in the x, y, or z plane and < 2 angular rotation about each axis.

### fALFF calculation

The ALFF maps of each voxel were assessed by applying band-pass filtering in the range of 0.01–0.08 Hz by using DPABI. Subsequently, fALFF was derived by dividing the low-frequency power by the standard deviation of the unfiltered signal. To standardize the data, z-scores for fALFF were computed for each subject.

### Functional connectivity analysis

In consideration of the primary fALFF outcomes and the reported widespread and significant alterations observed in the basal ganglia (BG) region, we conducted a seed-based FC analysis. Four regions of interest (ROI) were delineated, including the bilateral caudate nuclei and bilateral putamen. The reference time series for each seed were generated by averaging the time series of all voxels within the respective seed region. Pearson’s correlation coefficients were subsequently calculated between the time course of each seed region and those of all other voxels in the brain. These correlation coefficients were then transformed into *z*-values using Fisher’s r-to-z transformation to attain normality, resulting in seed-to-voxel FC maps for each subject.

### Statistical analysis

The demographic and clinical characteristics of participants across different groups were analyzed using SPSS 24.0 software. The normal distribution of data was assessed with the Shapiro–Wilk test. Sex distribution was examined using the chi-square test. For continuous variables between the two groups, independent sample t-tests or Mann–Whitney U-tests were employed, depending on the data distribution. Among the three groups, including age, education level, and MoCA score, one-way ANOVA or Kruskal–Wallis tests were used, with *post hoc* Bonferroni tests applied. A *p*-value <0.05 was considered statistically significant.

A one-way ANOVA design model was employed using SPM12 among the three groups (MSA-P, PD, and HC), with age, sex, and education level as covariates to identify significant differences in the z-value maps (voxel-level *p* < 0.05, cluster-level p < 0.05, uncorrected). These significant differences were then extracted as masks. Subsequently, *post hoc* two-sample t-tests were conducted between each pair of the three groups, with age, sex, and education level considered as covariates. When comparing PD with MSA-P, medication usage was also included as a covariate. The significance threshold at the voxel level was set at *p* < 0.001, with family-wise error (FWE) correction for multiple comparisons at the cluster level (*p* < 0.05). Additionally, a less stringent threshold of *p* < 0.005 at the voxel level, corrected by FWE correction at the cluster level (*p* < 0.05), was applied.

The diagnostic accuracy of fALFF in regions with differences was further assessed through receiver operating characteristic (ROC) curve analyses. Accuracy for differential diagnosis was quantified using the area under the curve (AUC) and values of sensitivity and specificity. A significance level of *p* < 0.05 was considered statistically significant.

## Results

### Clinical characteristics

The demographic and clinical characteristics of the study participants are presented in Table 1. The total sample included 85 individuals, with 24 diagnosed with MSA-P, 34 with PD, and 27 HC. No notable differences were found among the three groups (MSA-P, PD, and HC) in terms of age, sex and education years and MoCA scores. Furthermore, there were no significant variations in the disease duration and assessment scores (HAMD and HAMA scores) between the MSA-P and PD groups.

**Table 1:** Demographic and clinical characteristics.

Characteristics	MSA-P	PD	HC	*P*
Number, n	24	34	27	–
Age, years	55.04 ± 6.11	51.50 ± 8.40	50.26 ± 6.85	0.061
Sex, male/female	15/9	17/17	11/16	0.299
Education, years	12.96 ± 3.43	11.97 ± 3.57	12.33 ± 2.66	0.675
MoCA score	25.54 ± 1.93	25.35 ± 2.74	26.73 ± 1.80	0.070
Disease duration, years	1.67 ± 0.64	1.50 ± 0.79	–	0.659
HAMD score	4.83 ± 3.36	3.38 ± 2.37	–	0.544
HAMA score	5.25 ± 3.71	4.97 ± 3.44	–	1.000
UMSARS-I score	10.17 ± 2.84	–	–	–
UMSARS-II score	14.46 ± 3.72	–	–	–
UPDRS-III score	-	26.15 ± 8.78	–	–
H &Y stage	-	1.56 ± 0.47	–	–
LEDD, mg/d	352.26 ± 208.81	290.56 ± 196.96	–	0.193

MSA-P, parkinsonian variant of multiple system atrophy; PD, Parkinson’s Disease; HC, healthy controls; MoCA, the Montreal Cognitive Assessment; HAMD, the 24-item Hamilton Depression Rating Scale; HAMA, the Hamilton Anxiety Rating Scale; UMSARS, Unified Multiple System Atrophy Rating Scale; UPDRS, Unified PD Rating Scale; H&Y, Hoehn & Yahr; LEDD, levodopa equivalent daily dose.

### Regional function

**Figure 1:**
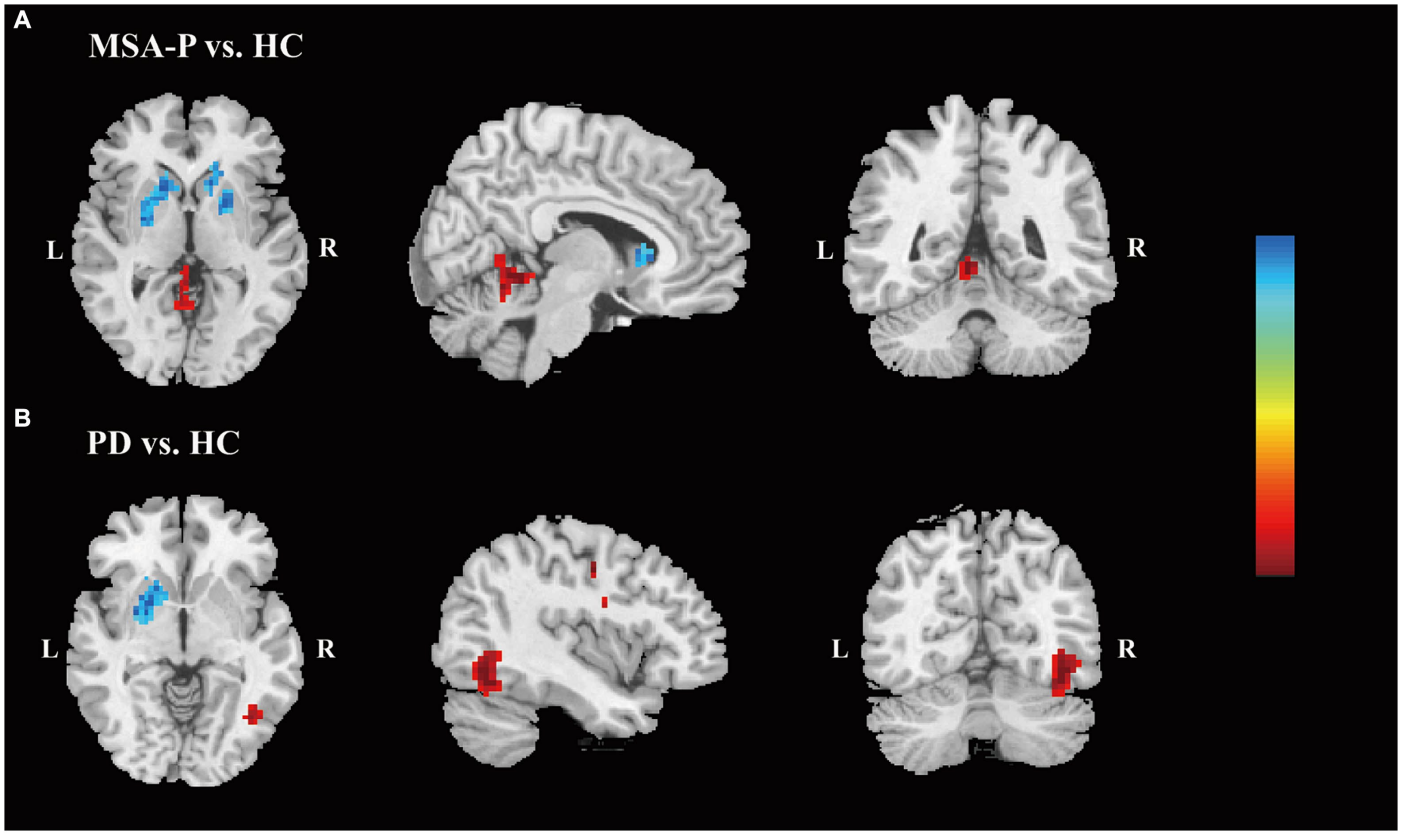
Differences of the fALFF values. Differences in the fALFF values are shown between MSA-P patients and HC **(A)** and between PD patients and HC **(B)**. (*p* < 0.005, family-wise error-corrected). The color bar indicates z value. The warm colors indicate that the *z* values of the MSA-P (A) or PD **(B)** groups are higher than those of the HC group, while the cool colors indicate that the z-values are lower. Abbreviations: fALFF, fractional amplitude of low-frequency fluctuation; MSA-P, parkinsonian variant of multiple system atrophy; HC, healthy controls; PD, Parkinson’s Disease; L, left; R, right.

Compared to HC, the MSA-P group exhibited reduced fALFF in the left putamen and caudate, along with an increased fALFF value in the left superior cerebellum (Supplementary Table S1). Additionally, the PD group showed decreased fALFF in the left putamen (Supplementary Table S1). With a slightly loosened significance threshold, MSA-P group displayed reduced fALFF in both the left and right putamen and caudate, coupled with an elevated fALFF value in the left cerebellum (Figure 1A and Supplementary Table S1). Regarding the PD group, it exhibited decreased fALFF in the left BG and left medial superior frontal gyrus (mSFG), as well as increased fALFF in the right inferior occipital gyrus (IOG), fusiform gyrus (FFG), and inferior temporal gyrus (ITG) (Figure 1B and Supplementary Table S1).

### *Post-hoc* analysis

**Figure 2:**
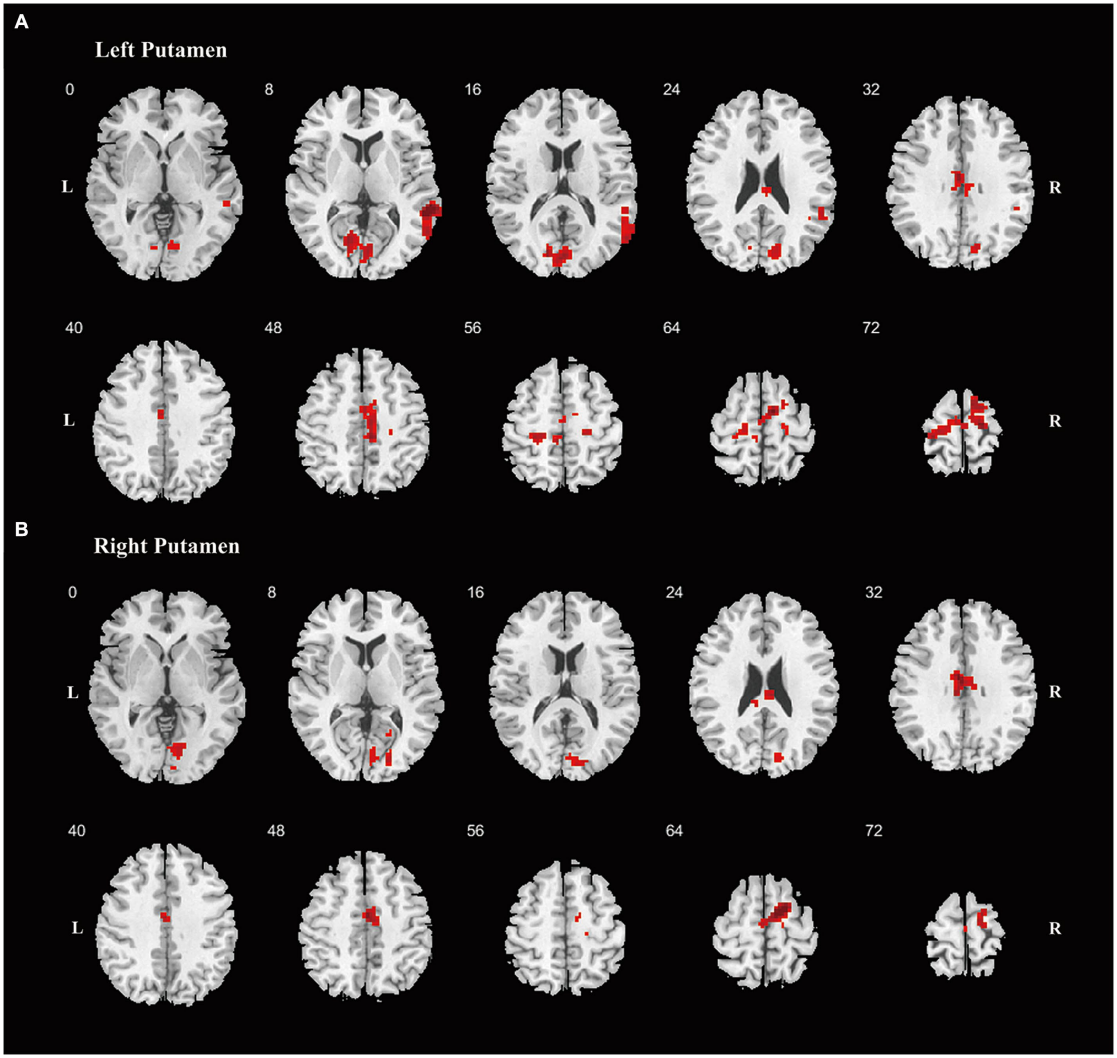
Differences of the FC. Differences of FC are shown between PD and MSA-P patients, using striatum regions as seeds, including left putamen **(A)**, right putamen **(B)**. Increased FC was shown in red color. Abbreviations: FC, functional connectivity; PD, Parkinson’s Disease; MSA-P, parkinsonian variant of multiple system atrophy; L, left; R, right.

For the FC analysis, focusing on the FC between four ROIs encompassing the bilateral caudate nuclei and bilateral putamen and the other voxels in the brain, the results indicated that, compared to the HC, MSA-P patients displayed reduced FC between the left putamen and the right BG, while PD patients exhibited increased FC between bilateral putamen and various regions (Supplementary Table S2). Compared to MSA-P group, the PD group exhibited increased FC between the left putamen and the right precentral gyrus and right supplementary motor area (SMA) (Supplementary Table S2). When the significance threshold was slightly relaxed, the PD group also exhibited increased FC between the left putamen and the left paracentral lobule and right middle temporal gyrus, the right putamen and right SMA, as well as bilateral putamen and several regions, including the bilateral median cingulate and paracingulate gyrus, bilateral calcarine fissure, and surrounding cortex, compared to the MSA-P group (Figure 2 and Supplementary Table S2).

Based on the Z-values in the four ROI mentioned above, ROC analysis was performed to assess the ability of fALFF to distinguish among patients with MSA-P and PD and HC. It showed that fALFF of left caudate resulted in 79.2% sensitivity and 81.5% specificity, with an AUC of 0.838, to distinguish MSA-P patients and HC; 54.2% sensitivity and 97.1% specificity, with an AUC of 0.772, to distinguish MSA-P patients from PD (Table 2). And fALFF of left putamen resulted in 61.8% sensitivity and 85.2% specificity, with an AUC of 0.736, to distinguish PD patients and HC (Table 2).

**Table 2:** Discriminatory value of fALFF.

Diagnostic groups	Predictor	AUC	Sensitivity (%)	Specificity (%)
MSA-P vs. HC	Left caudate	0.838	79.2	81.5
	Right caudate	0.827	87.5	66.7
	Left putamen	0.798	79.2	74.1
	Right putamen	0.827	70.8	81.5
PD vs. HC	Left caudate	0.708	94.1	51.9
	Right caudate	0.685	79.4	63.0
	Left putamen	0.736	61.8	85.2
	Right putamen	0.655	91.2	40.7
MSA-P vs. PD	Left caudate	0.772	54.2	97.1
	Right caudate	0.678	62.5	70.6
	Left putamen	0.566	50.0	70.6
	Right putamen	0.680	87.5	50.0

fALFF, fractional amplitude of low-frequency fluctuation; MSA-P, parkinsonian variant of multiple system atrophy; PD, Parkinson’s Disease; HC, healthy controls; AUC, area under the curve.

## Discussion

Our fMRI investigation, focusing on spontaneous brain activity and connectivity patterns, revealed distinct alterations between MSA-P and PD at early stage. MSA-P patients exhibited reduced fALFF in the left putamen and caudate, accompanied with increased activity in the left superior cerebellum. PD patients, in contrast, displayed decreased fALFF in the left putamen. Moreover, PD patients demonstrated increased FC between bilateral putamen and specific regions, including the right precentral gyrus and SMA. Conversely, MSA-P patients showed reduced FC between the left putamen and the right BG. Notably, ROC curve analysis underscored the diagnostic potential of fALFF, particularly in regions like the left caudate and putamen, offering promise for fALFF as an indicator to differentiate between MSA-P and PD.

Various evidence suggest that changes in brain function may occur before noticeable brain atrophy, particularly in the initial stage of the disease (Perovnik et al., 2023). The detected alterations in fALFF within specific brain regions provided valuable insights into the distinctive neurobiological mechanisms associated with MSA-P and PD. The neuropathological features of MSA contributing to parkinsonian symptoms include widespread glial cytoplasmic inclusions containing fibrillar and modified α-synuclein in the striatonigral region (Campese et al., 2021). Both MSA-P and PD initially involve degeneration restricted to the substantia nigra before putamen and followed by affecting the caudate (Poewe et al., 2022). Our study revealed a significant reduction in fALFF in the caudate nucleus of MSA-P patients, in addition to the significant decrease in fALFF observed in the putamen in both MSA-P and PD patients. These findings may suggest a different progression pattern involving the caudate nucleus in MSA-P compared to PD. This similarity in results has been supported by previous positron emission tomography (PET) study, which indicated relatively preserved function of the caudate nucleus in PD patients compared to MSA-P patients (Otsuka et al., 1997). Furthermore, PET scans in PD patients have shown progressive dopaminergic loss in the putamen, with asymmetry between affected striatal regions (Politis et al., 2017). Correspondingly, our investigation revealed decreased fALFF values in the putamen of PD patients, in line with earlier ALFF studies (Wu et al., 2015; Hou et al., 2022). Notably, with a slightly relaxed significance threshold, alterations in fALFF were observed in the bilateral BG of MSA-P patients, whereas in PD patients, only the left BG exhibited functional alterations. This pattern underscored the prominent unilateral or asymmetrical onset signs in PD patients (Poewe et al., 2017). Overall, the observed reduction in fALFF in the BG in our study, suggests that fALFF may offer valuable insights for identifying MSA-P and PD in the early stage.

Moreover, the observed alterations in fALFF within the superior cerebellum in MSA-P patients and various cortical regions in PD patients in our study were noteworthy, as they corresponded to some extent with the pathophysiological changes observed in the two diseases. Despite MSA-P patients predominantly presenting with parkinsonian symptoms, alongside functional changes in the striatal region, the involvement of the cerebellum was also significant. The role of the cerebellum has received growing attention, encompassing both motor control and cognitive processes, with the anterior cerebellar lobes specifically linked to motor activities (Grimaldi and Manto, 2012). In a study enrolling both MSA-P and MSA-C patients indicated that patients with MSA-P also had abnormal cerebellar volume, with longitudinal follow-up revealing significant structural changes in the cerebellum (Vemuri et al., 2022). Conversely, while another study investigating ALFF including both MSA-P and MSA-C patients found higher ALFF value in the cerebellum (Wang et al., 2017), our investigation, focusing on fALFF and solely MSA-P patients, demonstrated elevated value in the cerebellum, further emphasizing altered cerebellar function at an early stage in MSA-P patients. In addition, we observed altered fALFF values in IOG, FFG, ITG and mSFG in PD patients. Alterations in spontaneous brain activity in the temporo-occipital cortex are common in PD patients. Previous studies reported increased brain activity in the occipital cortex (Wang et al., 2017; Wang et al., 2020) and temporal cortex (Wang et al., 2017; Wang et al., 2023; Wang et al., 2020; Wu et al., 2015), which were consistent with our findings, whereas, some studies have documented decreased activity in the occipital cortex (Hou et al., 2022; Wang et al., 2023; Wu et al., 2015). Given its role as a visual processing center, deficits in the occipital cortex have been associated with impaired visual cues for locomotion (Sage and Almeida, 2010), and the presence of non-motor symptoms like visual hallucinations has been correlated with functional alterations in both the temporal and occipital cortex (Sage and Almeida, 2010; Ibarretxe-Bilbao et al., 2011). The elevated fALFF observed in our study may indicate heightened neuronal activity in the early stage, possibly serving as a compensatory mechanism to maintain both motor and non-motor functions. With the gradual disease progression, this compensatory effect may diminish or disappear at the advanced stage. Generally, these findings confirmed the functional alterations observed across various regions during the early stage of the two conditions, and highlighted the differences in pathological and compensatory mechanisms between the two conditions.

The BG has been considered an independent brain region implicated in parkinsonism, but they also closely collaborated with the cortex to support motor and non-motor functions (Caligiore et al., 2019). The alterations in FC patterns underscore the distinct cortical–subcortical circuitry involved in the two conditions. Compared to MSA-P patients, PD patients exhibited increased FC between the putamen and motor-related regions such as the precentral gyrus and SMA. Given that FC involving precentral gyrus and SMA are crucial for motor control and action sequencing (Caligiore et al., 2016), dysfunction in these regions may contribute to observed motor symptoms in parkinsonism. Consequently, the elevated FC between BG and precentral gyrus /SMA in PD patients, relative to MSA-P patients, could suggest a more effective motor compensatory mechanism. Additionally, under a relaxed threshold, PD patients displayed increased FC between the putamen and other cortical regions, including the paracentral lobule and cingulum gyrus, as well as the temporal and occipital gyrus, which are associated with both motor and non-motor symptoms. The increased FC may also reflect enhanced compensatory mechanisms within the cortico-striatal circuitry involved in symptom mediation in PD patients. Notably, MSA-P patients did not exhibit increased FC, and the reduced FC between the left putamen and the right BG in MSA-P patients corresponded to the disorder’s characteristic of more severe symptoms compared to PD. Thus, the distinct FC patterns between BG and cortex differentiated MSA-P from PD, reflecting diverse compensatory mechanisms and the severity of the diseases. The alterations in FC patterns also highlight the potential clinical value of these measures in differentiating between MSA-P and PD. Early identification of these FC patterns might guide targeted and customized management strategies.

The diagnostic potential of fALFF, validated through ROC analysis, emphasized its utility as a promising marker for distinguishing the two neurodegenerative disorders. The fALFF value of putamen demonstrated acceptable discriminatory ability in identifying PD patients and HC in our study, consistent with prior research, which showed that classification models incorporating putamen imaging features may effectively distinguish PD patients from HC (Hou et al., 2022). Our findings also underscored the utility of fALFF values in the caudate nucleus for distinguishing MSA-P from PD. These distinct fALFF patterns emphasize significant clinical implications, potentially aiding in early diagnosis and supporting the development of tailored treatment approaches for MSA-P and PD. While the AUC values of fALFF in BG were acceptable, further validation in larger cohorts are warranted and future investigations could explore models that combine fMRI imaging features with other MRI sequences or clinical variables to enhance diagnostic accuracy.

Limitations of the study include a relatively small sample size and its cross-sectional design. Future research would benefit from a larger sample size and longitudinal designed studies to validate the findings of the current study. Despite efforts to control for confounding variables like neuropsychiatry and cognition, other factors such as cumulative drug effects may still have influenced the results. While medication use was inevitable for accurate diagnosis, strict measures were taken to minimize its impact by requiring patients to abstain from medication for at least 12 h before MRI examinations.

## Conclusion

Our research enhanced understanding of the underlying mechanisms of MSA-P and PD, as evidenced by alterations in fALFF and FC patterns. These findings underscore the potential of fALFF as a diagnostic marker for distinguishing MSA-P from PD.

## Data availability statement

The raw data supporting the conclusions of this article will be made available by the authors, without undue reservation.

## Ethics statement

The studies involving humans were approved by the Ethics Committee of West China Hospital, Sichuan University (No. 2015–236). The studies were conducted in accordance with the local legislation and institutional requirements. The participants provided their written informed consent to participate in this study.

## Author contributions

SW: Conceptualization, Data curation, Formal analysis, Funding acquisition, Investigation, Methodology, Project administration, Resources, Supervision, Validation, Visualization, Writing – original draft, Writing – review & editing. YX: Conceptualization, Data curation, Formal analysis, Investigation, Methodology, Project administration, Resources, Software, Supervision, Validation, Writing – original draft, Writing – review & editing. YH: Conceptualization, Data curation, Formal analysis, Funding acquisition, Investigation, Methodology, Project administration, Resources, Software, Supervision, Validation, Visualization, Writing – review & editing. CL: Conceptualization, Data curation, Formal analysis, Investigation, Methodology, Project administration, Resources, Software, Supervision, Validation, Visualization, Writing – review & editing. LZ: Data curation, Resources, Writing – review & editing. RO: Data curation, Resources, Writing – review & editing. QW: Data curation, Resources, Writing – review & editing. JuL: Data curation, Resources, Writing – review & editing. TY: Data curation, Resources, Writing – review & editing. NC: Data curation, Resources, Writing – review & editing. QJ: Data curation, Resources, Writing – review & editing. XZ: Data curation, Resources, Writing – review & editing. JiL: Data curation, Resources, Writing – review & editing. HS: Writing – review & editing, Conceptualization, Data curation, Formal analysis, Funding acquisition, Investigation, Methodology, Project administration, Resources, Software, Supervision, Validation, Visualization.
