# Supplementary material for: Comparison of spontaneous brain activity in distinguishing parkinsonian variant of multiple system atrophy from Parkinson’s disease at an early stage
Source: Front Aging Neurosci. 2024 Aug 29;16:1427991. doi: 10.3389/fnagi.2024.1427991 (PMC11390528; doi:10.3389/fnagi.2024.1427991)
Supplement: Supplementary file 1 [file Table_1.docx]

| **Table S1: Difference in fALFF** | | | | | | | | |
| --- | --- | --- | --- | --- | --- | --- | --- | --- |
|  | **Cluster size** | **P value** | **brain region** | **clsuter size** | **T value** | **MNI coordinate (x, y, z)** | | |
| voxel level p < 0.001, cluster level p-FWE < 0.05 | | |  |  |  |  |  |  |
| MSA-P<HC |  |  |  |  |  |  |  |  |
| Cluster 1 | 42 | 0.01 | Putamen_L | 23 | 4.42 | -15 | 15 | -3 |
|  |  |  | Caudate_L | 15 | 4.32 | -9 | 18 | 6 |
| MSA-P>HC |  |  |  |  |  |  |  |  |
| Cluster 1 | 30 | 0.04 | Cerebelum_4_5_L | 25 | 4.58 | -6 | -48 | -3 |
| PD<HC |  |  |  |  |  |  |  |  |
| Cluster 1 | 55 | 0.00 | Putamen_L | 36 | 4.82 | -24 | 9 | -6 |
| voxel level p < 0.005, cluster level p-FWE < 0.05 | | |  |  |  |  |  |  |
| MSA-P<HC |  |  |  |  |  |  |  |  |
| Cluster 1 | 133 | 0.00 | Putamen_L | 72 | 4.42 | -15 | 15 | -3 |
|  |  |  | Caudate_L | 35 | 4.32 | -9 | 18 | 6 |
| Cluster 2 | 87 | 0.01 | Putamen_R | 24 | 3.93 | 18 | 3 | 9 |
|  |  |  | Caudate_R | 19 | 4.31 | 12 | 18 | -3 |
| MSA-P>HC |  |  |  |  |  |  |  |  |
| Cluster 1 | 74 | 0.03 | Cerebelum_4_5_L | 50 | 4.58 | -6 | -48 | -3 |
| PD<HC |  |  |  |  |  |  |  |  |
| Cluster 1 | 144 | 0.00 | Putamen_L | 84 | 4.38 | -24 | 9 | -6 |
|  |  |  | Pallidum_L | 19 | 4.82 | -24 | 9 | -6 |
|  |  |  | Caudate_L | 12 | 4.21 | -12 | 9 | 3 |
| Cluster 2 | 87 | 0.01 | Frontal_Sup_Medial_L | 58 | 3.90 | -3 | 45 | 18 |
| PD>HC |  |  |  |  |  |  |  |  |
| Cluster 1 | 80 | 0.00 | Occipital_Inf_R | 16 | 4.27 | 39 | -63 | -15 |
|  |  |  | Fusiform_R | 21 |  |  |  |  |
|  |  |  | Temporal_Inf_R | 18 |  |  |  |  |
| Abbreviations: fALFF, fractional amplitude of low-frequency fluctuation; MSA-P, parkinsonian variant of multiple system atrophy; PD, Parkinson's Disease; HC, healthy controls; MNI, Montreal Neurological Institute; FWE, family-wise error; L, left; R, right. | | | | | | | | |

| **Table S2: Difference in FC using striatum regions as seeds** | | | | | | | | |
| --- | --- | --- | --- | --- | --- | --- | --- | --- |
| **Seed area** | **Cluster size** | **P value** | **Connected region** | **clsuter size** | **T value** | **MNI coordinate (x, y, z)** | | |
| Left putamen |  |  |  |  |  |  |  |  |
| voxel level p < 0.001, cluster level p-FWE < 0.05 | |  |  |  |  |  |  |  |
| PD>HC |  |  |  |  |  |  |  |  |
| Cluster 1 | 82 | 0.01 | Precentral_R | 52 | 4.16 | 18 | -21 | 72 |
|  |  |  | Postcentral_R | 21 | 3.85 | 30 | -27 | 63 |
| MSA-P<PD |  |  |  |  |  |  |  |  |
| Cluster 1 | 72 | 0.03 | Precentral_R | 21 | 3.77 | 15 | -18 | 72 |
|  |  |  | Supp_Motor_Area_R | 22 | 3.95 | 9 | -9 | 75 |
| voxel level p < 0.005, cluster level p-FWE < 0.05 | |  |  |  |  |  |  |  |
| MSA-P<HC |  |  |  |  |  |  |  |  |
| Cluster 1 | 300 | 0.00 | Putamen_R | 81 | 4.29 | 18 | 6 | -6 |
|  |  |  | Caudate_R | 39 |  |  |  |  |
|  |  |  | Pallidum_R | 27 |  |  |  |  |
| PD>HC |  |  |  |  |  |  |  |  |
| Cluster 1 | 211 | 0.01 | Frontal_Inf_Oper_L | 66 | 4.38 | -51 | 9 | 6 |
|  |  |  | Insula_L | 30 | 3.46 | -42 | 18 | 3 |
|  |  |  | Frontal_Inf_Orb_L | 20 |  |  |  |  |
| Cluster 2 | 203 | 0.01 | Precentral_R | 122 | 4.16 | 18 | -21 | 72 |
|  |  |  | Postcentral_R | 47 | 3.85 | 30 | -27 | 63 |
| MSA-P<PD |  |  |  |  |  |  |  |  |
| Cluster 1 | 618 | 0.00 | Supp_Motor_Area_R | 167 | 4.06 | 9 | -6 | 63 |
|  |  |  | Cingulum_Mid_L | 48 | 3.99 | -3 | -9 | 36 |
|  |  |  | Paracentral_Lobule_L | 74 |  |  |  |  |
|  |  |  | Cingulum_Mid_R | 56 |  |  |  |  |
|  |  |  | Precentral_R | 46 |  |  |  |  |
| Cluster 2 | 223 | 0.01 | Temporal_Mid_R | 124 | 4.80 | 57 | -42 | 9 |
|  |  |  | SupraMarginal_R | 36 | 3.47 | 54 | -48 | 27 |
| Cluster 3 | 321 | 0.00 | Cuneus_L | 51 | 3.55 | -15 | -78 | 15 |
|  |  |  | Cuneus_R | 84 | 3.55 | 12 | -72 | 30 |
|  |  |  | Calcarine_L | 106 | 3.51 | -15 | -69 | 9 |
|  |  |  | Calcarine_R | 32 |  |  |  |  |
| Right putamen |  |  |  |  |  |  |  |  |
| voxel level p < 0.001, cluster level p-FWE < 0.05 | |  |  |  |  |  |  |  |
| PD>HC |  |  |  |  |  |  |  |  |
| Cluster 1 | 125 | 0.00 | Precentral_R | 79 | 4.31 | 24 | -24 | 66 |
|  |  |  | Postcentral_R | 18 |  |  |  |  |
|  |  |  | Supp_Motor_Area_R | 15 |  |  |  |  |
| voxel level p < 0.005, cluster level p-FWE < 0.05 | |  |  |  |  |  |  |  |
| PD>HC |  |  |  |  |  |  |  |  |
| Cluster 1 | 425 | 0.00 | Precentral_R | 167 | 4.31 | 24 | -24 | 66 |
|  |  |  | Supp_Motor_Area_R | 90 |  |  |  |  |
|  |  |  | Postcentral_R | 61 |  |  |  |  |
| Cluster 2 | 212 | 0.01 | Occipital_Mid_L | 101 | 3.77 | -36 | -69 | 0 |
|  |  |  | Temporal_Mid_L | 25 |  |  |  |  |
| Cluster 3 | 353 | 0.00 | Paracentral_Lobule_R | 53 | 3.73 | 3 | -33 | 63 |
|  |  |  | Paracentral_Lobule_L | 81 | 3.63 | -12 | -36 | 72 |
|  |  |  | Postcentral_L | 47 |  |  |  |  |
|  |  |  | Precuneus_L | 45 |  |  |  |  |
| MSA-P<PD |  |  |  |  |  |  |  |  |
| Cluster 1 | 365 | 0.00 | Supp_Motor_Area_R | 105 | 4.90 | 15 | -3 | 66 |
|  |  |  | Cingulum_Mid_L | 64 | 4.04 | -6 | -9 | 36 |
|  |  |  | Cingulum_Mid_R | 40 |  |  |  |  |
| Cluster 2 | 168 | 0.04 | Calcarine_R | 78 | 3.33 | 18 | -84 | 12 |
|  |  |  | Lingual_R | 27 | 3.22 | 12 | -72 | 0 |
|  |  |  | Cuneus_R | 36 |  |  |  |  |
|  |  |  | Calcarine_L | 16 |  |  |  |  |
| Abbreviations: FC, functional connectivity; MSA-P, parkinsonian variant of multiple system atrophy; PD, Parkinson's Disease; HC, healthy controls; MNI, Montreal Neurological Institute; FWE, family-wise error; L, left; R, right. | | | | | | | | |
